# Development of 2,3-butanedione-sensing element using Pt-nanoparticle-decorated tin oxide for health applications

**DOI:** 10.3389/fchem.2025.1575479

**Published:** 2025-06-18

**Authors:** A. Fotia, A. Malara, L. Bonaccorsi, A. Macario, A. Gnisci, P. Frontera

**Affiliations:** ^1^ Department of Engineering, University of Messina, Messina, Italy; ^2^ Department of Civil, Energy, Environmental and Materials Engineering, Mediterranean University of Reggio Calabria, Reggio Calabria, Italy; ^3^ Department of Environmental Engineering, University of Calabria, Cosenza, Italy; ^4^ Malvern Panalytical S.r.l, Lissone, Monza-Brianza, Italy

**Keywords:** 2,3-butanedione, sensing layer, nanoparticles, platinum doping, health device

## Abstract

An efficient, reliable, and cost-effective sensor of 2,3-butanedione, an organic compound with a distinctive buttery aroma, is here developed through the production of a sensible layer based on a tin oxide semiconductor decorated with platinum nanoparticles. The resulting material is subjected to characterization and testing to evaluate its capability to sense 2,3-butanedione, which is known to be responsible for health risks upon inhalation, including the potential to cause lung disease, and to be detected in the breath of individuals with cystic fibrosis. The results demonstrate that the developed sensor provides high sensitivity and selectivity for 2,3-butanedione in specific experimental conditions, featuring advantages such as simplicity, portability, and rapid response. In addition, it shows great potential for on-site safety and health applications, providing a practical solution for real time monitoring.

## Introduction

The molecule 2,3-butanedione, also called diacetyl, is a naturally occurring organic compound with a distinct, buttery aroma and flavor. Its chemical formula is C_4_H_6_O_2_, and it belongs to the class of chemicals known as diketones. Often found in dairy products like butter and milk, 2,3-butanedione is also used as an artificial flavoring agent in various foods (such as microwave popcorn, baked goods, and candies), beverage products, and e-cigarettes (Schaeffer and Iannucci, 2014). Airborne exposure to volatile butter flavoring ingredients that include 2,3-butanedione is linked to case clusters of obstructive airways disease and the rare lung disease bronchiolitis obliterans among workers engaged in microwave popcorn production and flavoring manufacture (Iannucci and Carter, 2023).

This molecule which has been previously exclusively linked to lung injuries in factory workers producing microwave popcorn might play an important role in microbial lung infections in people with cystic fibrosis (Whiteson et al., 2014).

It is postulated by researchers that different types of the oral microorganism *Streptococcus* synthesize diacetyl through a fermentation mechanism. The chemical also possesses the ability to elicit deleterious effects on other bacteria that are frequently found in the lungs of individuals with cystic fibrosis. For instance, the interaction between *Pseudomonas aeruginosa* bacteria and diacetyl leads to the synthesis of harmful chemicals by the bacteria, perhaps contributing to the development of lung damage characteristic of cystic fibrosis. Subsequently, the compound 2,3-butanedione exhibits more elevated levels of detection in individuals affected by cystic fibrosis compared to those who are in a state of good health (Robroeks et al., 2010; Whiteson et al., 2014).

Traditionally, the process of disease diagnosis has predominantly relied on the analysis of blood and urine samples through the utilization of significant biomolecules—typically proteins or nucleic acids. In addition to genomics and proteomics, the subject of metabolomics is becoming prominent as it focuses on the direct analysis of metabolic pathways through the measurement of small-molecule metabolites. The volatile organic compounds (VOCs) mentioned are small-molecule metabolites that are present in the alveolar blood and may be found in the gas phase of exhaled breath. This leads to the development of a distinct metabolic pattern in the breath, permitting differentiation between individuals with a disease and those who are healthy (Bos et al., 2013).

Exhaled breath analysis is a field of research and clinical diagnostics that focuses on detecting and analyzing various volatile organic compounds, such as acetone, isoprene, and specific biomarkers to diagnose or monitor certain medical conditions (Kaloumenou et al., 2022). These compounds can provide insights into conditions like diabetes, kidney disease, liver disease, and respiratory conditions such as asthma and chronic obstructive pulmonary disease. However, diacetyl is not a commonly measured compound in exhaled breath analysis for diagnostic purposes.

**FIGURE 1:**
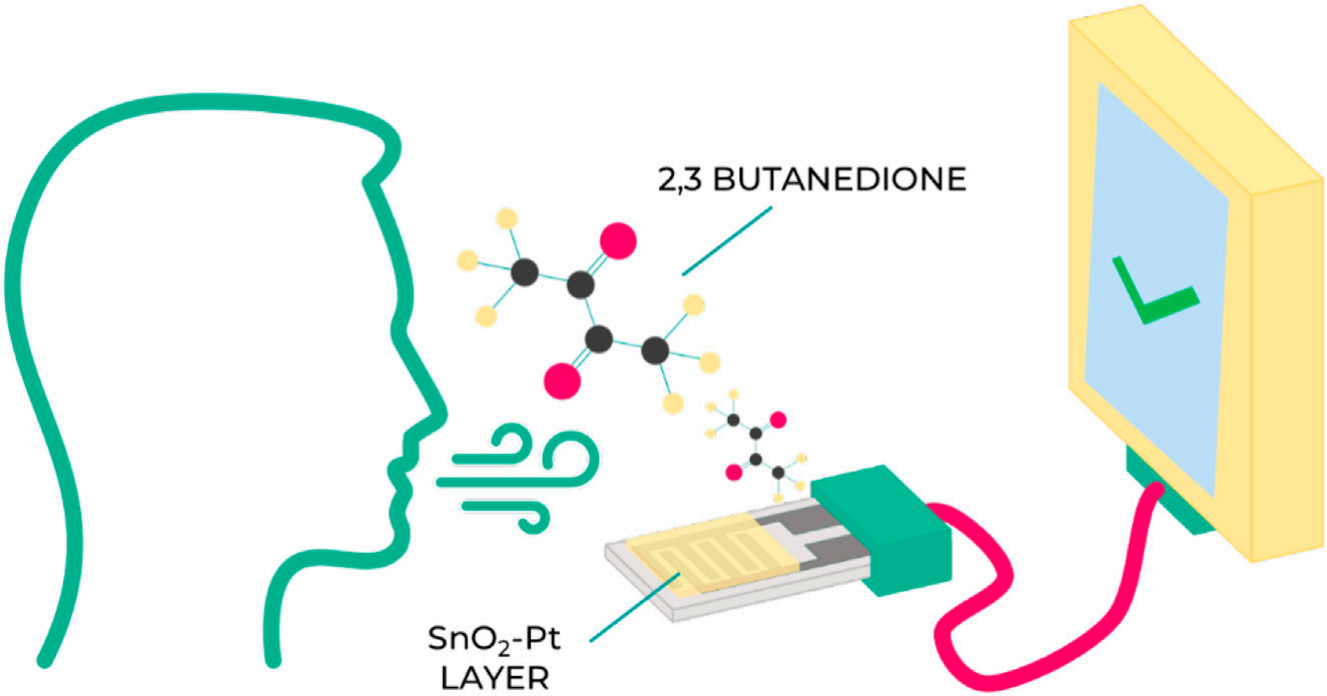
Visual summary of the research article.

The research proposed develops a sensing layer based on these findings that would act like a breath-analyzer and detect the presence of diacetyl (Figure 1). By regularly monitoring the presence of this molecule in their breath, fibrous cystic patients may gain an early detection signal indicating that an exacerbation is imminent, allowing them to take antibiotics to prevent it.

Previously (Gnisci et al., 2022), we investigated the production of an SnO_2_-based sensor to detect the concentration of 2,3-butanedione vapor in different operating atmospheres corresponding to the replications of typical alcoholic scenarios in industrial application. Generally speaking, tin dioxide, a representative wide-bandgap (3.6 eV) n-type semiconductor, offers great advantages in gas sensing owing to its quick response and good stability (Malara et al., 2018). However, pure SnO_2_-based sensors suffer from poor selectivity and harsh working temperatures in gas detection. Hence, one strategy to offer improved sensitivity, selectivity, stability, and lower operating temperatures is sensor doping or decorating with noble metals, metal oxide, or others (Liu et al., 2015; Pantò et al., 2018; Fotia et al., 2023).

In terms of element doping or decorating, metal oxide semiconductors are commonly produced and functionalized with other elements or noble metals such as Pd, Au, Rh, and especially Pt to enhance gas sensing performance (Frontera et al., 2016; Liu et al., 2017; Jeong et al., 2018; Qiu et al., 2018; Bonaccorsi et al., 2019; Cai et al., 2019; Li et al., 2019; Malara et al., 2019).

Different types of hierarchical three-dimensional SnO_2_ nanocomposites have been modified with Pt nanoparticles with enhanced sensing performance (Wang et al., 2013; 2016).

Recently, Xu et al. (2020) found that a single atom of Pt deposited on SnO_2_ film results in a highly enhanced sensor response and a decrease of the sensor working temperature. The literature highlights the ability of metallic nanoparticles to exhibit remarkable characteristics such as enhanced reactivity and improved electrical conductivity. These nanoparticles offer promising applications not only in sensing but across other fields, including medicine for targeted drug delivery, environmental science for pollution remediation, and electronics for developing more efficient devices, highlighting their transformative potential in addressing contemporary challenges (Huang et al., 2024; Li et al., 2025a; Li et al., 2025b; Su et al., 2023; Zhang et al., 2023).

To date, Pt-doped tin oxide has not been applied to detect 2,3-butanedione. We propose the synthesis, characterization, and testing of a gas sensor based on a platinum-doped tin oxide semiconductor that could be considered a desirable tool for on-site detection due to the advantages of simplicity, portability, cost-effectiveness, easy operation, and fast response to target molecules.

## Experimental

### Preparation of sensing materials

A hydrothermal method was utilized to synthesize SnO_2_ powder, following a procedure described elsewhere. Initially, SnCl_2_(II) was dissolved in ethanol at a concentration of 7.57 g/L to act as the tin oxide precursor. To ensure full dissolution of SnCl_2_, the mixture underwent sonication for 20 min. Subsequently, it was placed in a Teflon-lined stainless-steel autoclave at 200°C for 7 h. After that, the solution was cooled to room temperature. The resulting yellow solid was harvested via centrifugation, washed in ethanol to completely eliminate chloride ions, and lastly was dried overnight at 80°C.

The creation of SnO_2_-Pt involved using SnO_2_ as a support impregnated with platinum (3 wt% loading with respect to the tin oxide mass). This was achieved by impregnating a solution of chloroform and platinum (II) acetylacetonate into the SnO_2_ sample via incipient impregnation.

All samples before sensing test were reduced in hydrogen atmosphere at 150°C.

### Characterization

The crystalline structure of the materials was examined using a Panalytical Empyrean S-2 diffractometer that acquired XRD spectra *versus* rising temperatures from 25°C to 400°C using Cu–Kα radiation (1.54056 Å) at 40 kV and 40 mA. Step-scan mode was used to record patterns from 15° to 65° 2-theta angles in 0.02° increments and 5 s each step. Spectra were analyzed with the dedicated Highscore software.

The SEM-EDX investigation was conducted to gain insights into the morphological and compositional configurations of samples. A high-resolution scanning electron microscope, the Jeol JSM-7900F, which specializes in low vacuum measurements and investigations of low accelerating voltages, was used to conduct a thorough analysis. Using X-ray spectroscopy, the system was connected to a chemical microanalysis system (BRUKER Esprit). EDX analysis was utilized to examine the metal’s content and distribution; gathering at least 20 examination points for each sample at three different magnifications, 120 s were counted for all spectra acquisition. All samples yielded findings that were repeatable to less than 5%.

A transmission electron microscopy (TEM) investigation was carried out with the assistance of a JEOL 1400 Plus apparatus that maintained a voltage of 120 kV and had the capability of achieving a point-to-point resolution of 0.19 nm and a line resolution of 0.14 nm.

The porosity of the samples was determined by equilibrium adsorption and desorption isotherms of N_2_ at 77 K with a Micromeritics ASAP 2020 instrument. Before the analysis, all samples were pre-treated in vacuum condition at 200°C for 12 h. In order to determine the total surface area of the samples, the data collected were modeled using the BET equation.

### Sensor preparation and testing

**FIGURE 2:**
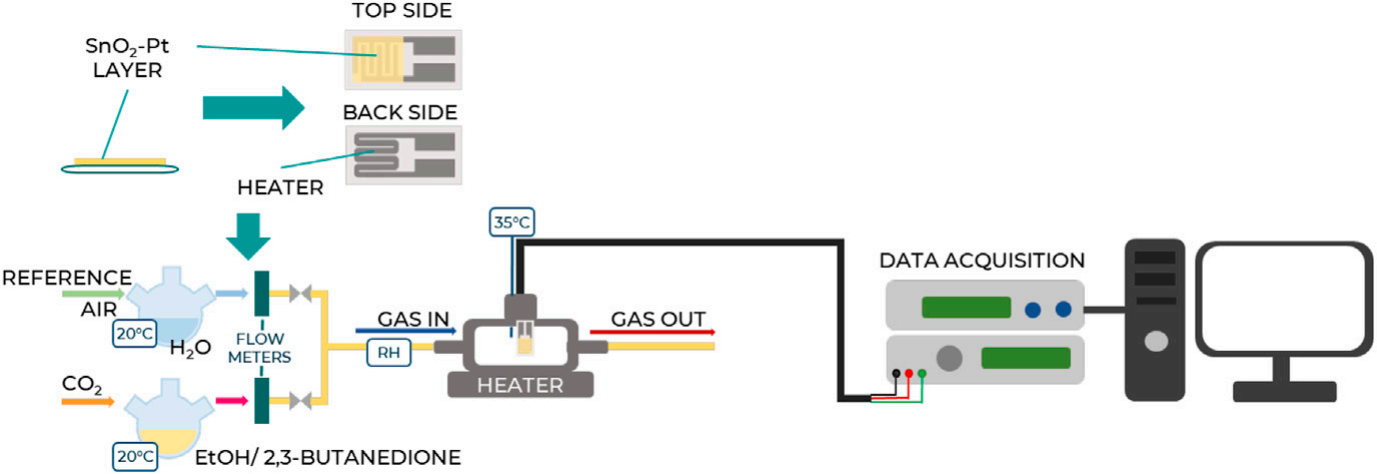
Schematic representation of the test apparatus showcasing the key components and setup for experimental testing.

The sensing oxide powder, SnO_2_-Pt, was mixed with ethanol and deposited as a paste on a commercial alumina planar substrate equipped with interdigitated Pt electrodes on the top and a heating element on the back. The sensor was positioned in a stainless-steel testing cell and stabilized in air at 400°C for 2 h prior to measurements. Tests were conducted under a total gas flow of 100 sccm using air as the reference gas and 2,3-butanedione vapor as the analyte target, obtained by bubbling CO_2_ in the liquid analyte maintained at the controlled temperature of 35°C. Indeed, the influence of the atmosphere composition was reported to be fundamental in 2,3-butanedione detection and monitoring, especially during the fermentation process (Portno, 1966). Different analyte concentrations were used, ranging from 0.5 to 4 mg/L. Alcoholic compounds, considered the most interfering species, were also tested using ethanol with two volume concentrations equal to 2.5% and 5.0% v/v. Gases were measured by computer-controlled mass flow meters, and humidity was set as 27%. The resistance data of the sensor were collected in four-point mode by an Agilent 34970A (Santa Clara, CA, United States) multimeter, while the sensor temperature was controlled by a dual-channel power supplier instrument (Agilent E3632A, Santa Clara, CA, United States). The scheme of the test apparatus is represented in Figure 2.

## Results and discussion

**FIGURE 3:**
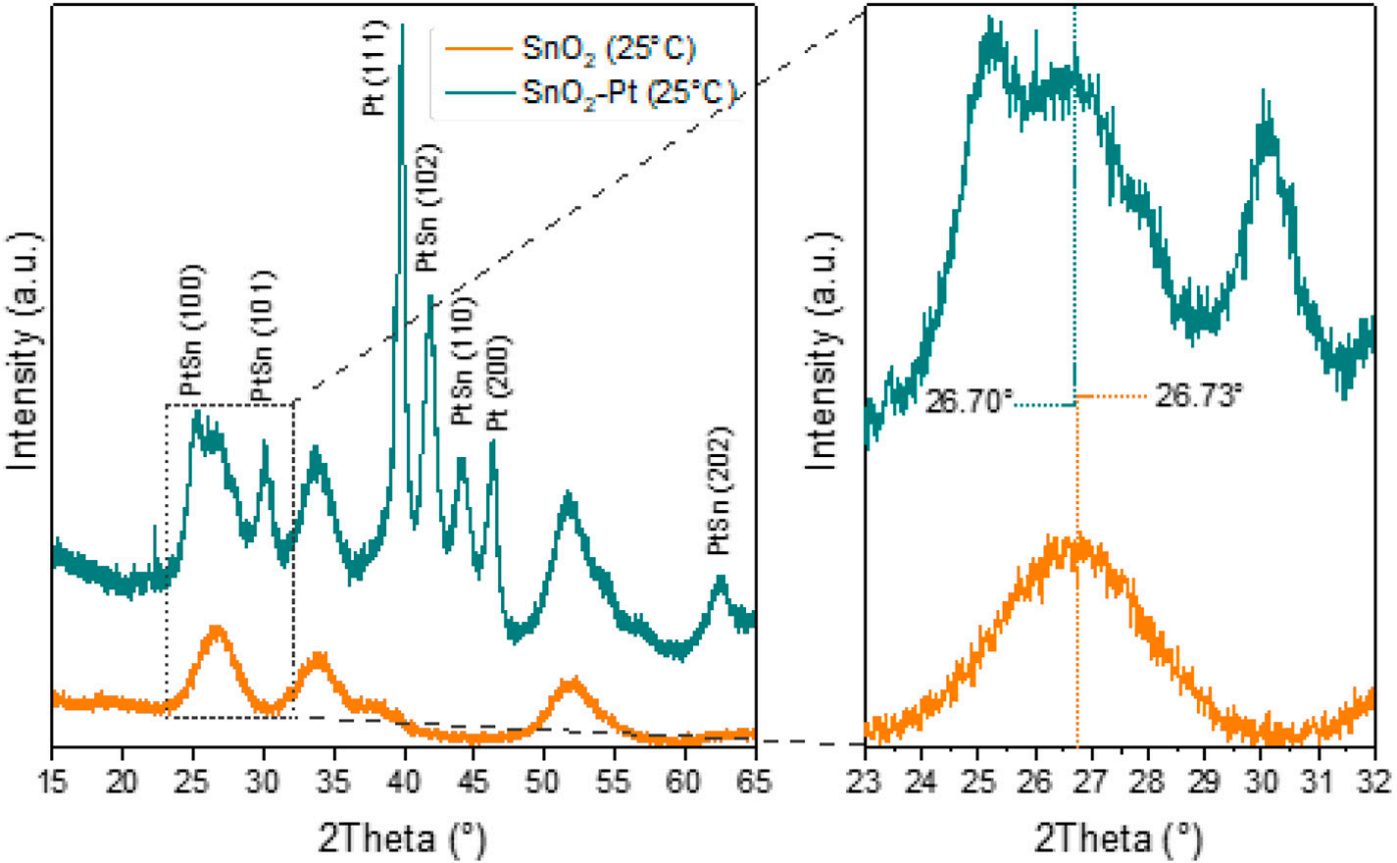
X-ray diffractograms of SnO_2_ and SnO_2_-Pt and related zoom in the 2-theta range 23°–38°.

The crystal structures of the synthesized tin oxide and Pt-decorated tin oxide were investigated by XRD analysis (Figure 3). The recorded patterns for the pure SnO_2_ show that the three intense diffractions peaks at 2θ = 26.7°, 33.9°, and 51.8° correspond to (110), (101), and (211) planes of SnO_2_, in according with JCPDS card No. 41-1445, confirming the tetragonal rutile crystal phase of the synthesized SnO_2_ nanomaterials. The most intense peak corresponds to (110) direction, showing the favored growth face and the predominant surface (110), which is the most stable surface in oxides with rutile structure. In fact, from an electrostatic point view, this surface is likely to be the most stable because it has the lowest density of dangling bonds (Manassidis et al., 1995). Consequently, the SnO_2_ structure exhibits elevated surface energy, resulting in enhanced oxygen absorption and subsequently improving the gas sensing performance of SnO_2_. Broadening the XRD peaks of SnO_2_ indicates that the crystallites in the material are small. This is because in a crystalline material with larger and more perfect crystallites, the lattice spacing (d-value) tends to be more uniform across the sample. As crystallites become smaller, the variations in d-values become more significant, leading to broader diffraction peaks (Langford et al., 2000).

The real content of platinum loaded into tin oxide is very close to nominal; in fact, the EDX analysis revealed a mean value of 3 ± 0.02 w%.

After Pt loading, in addition to SnO_2_ peaks (JCPDS no. 04-0802), two very high-intensity peaks related to Pt also appeared in the pattern at 2θ = 39.4° and 43.5°, which are assigned as Pt (111) and (200), respectively (Liu et al., 2017) (Figure 3).

The presence of these Pt peaks clearly revealed that the Pt is successfully distributed into the lattices of SnO_2_ support. Furthermore, a slight shift in the X-ray diffraction pattern of tin oxide toward higher angles was observed for the peak at 26.70°, so the platinum doping could modify the electronic properties of tin oxide and influence how X-rays interact with the material and lead to changes in the diffraction pattern (Gaskov et al., 2020).

**FIGURE 4:**
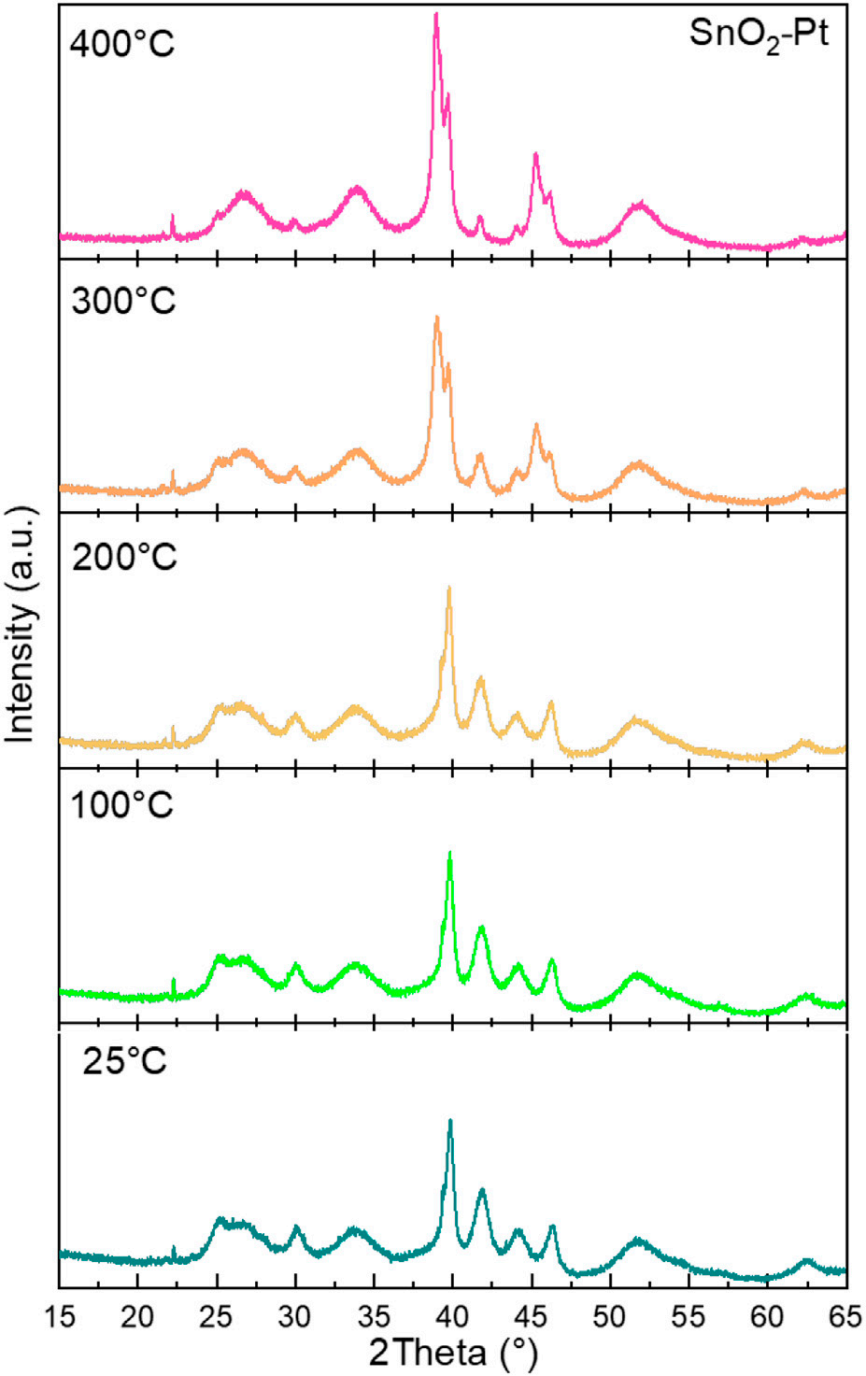
X-Ray diffractograms of SnO_2_-Pt *versus* temperature.

Since the annealing of the oxide onto the substrate device is always carried out at a high temperature, the effect of temperature on the crystal structure is also worthy of attention (Wang et al., 2013). Therefore, the SnO_2_-Pt was prepared at calcination temperatures varying from 25°C to 400°C; their XRD patterns are presented in Figure 4. The stability of the bare support has already been demonstrated (Liu et al., 2015), and the decorated support at every temperature exhibits all the characteristic diffraction peaks of SnO_2_-Pt, indicating the thermal stability of the crystalline materials.

**FIGURE 5:**
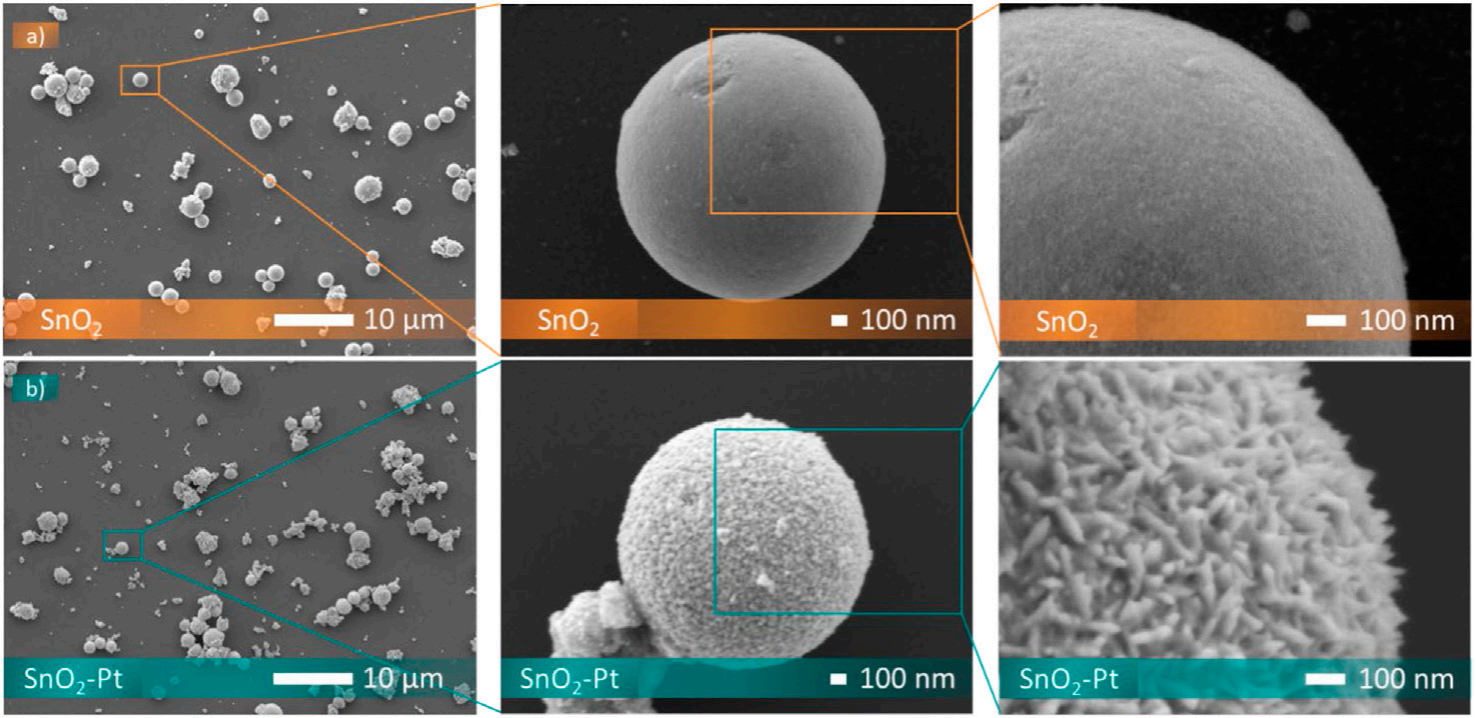
SEM images at different magnifications of SnO_2_ (a) and SnO_2_-Pt (b).

As shown in Figure 5, the particles of SnO_2_ are composed of spheres not interlinked to each other, with a narrow size distribution scale of a mean diameter of 1.4 ± 0,2 microns. This uniformity is indicative of controlled synthesis, and the obtaining of spherical morphology offers properties such as high surface area-to-volume ratio and efficient packing in the sensing layer. Moreover, the spherical particles of tin oxide exhibit smooth, dense, and uniform surfaces without sharp edges or irregularities.

The platinum doping does not affect the spherical morphologies, probably because the concentration of platinum dopants is relatively low and the impact on the overall nanoparticle morphology may be minimal. At lower dopant concentrations, the influence on particle shape is subdued, as already observed for the rod-like (Nam et al., 2019), hollow microsphere (Bizhou et al., 2018) and nanofiber (Jung and Jang, 2022) morphologies.

From high-magnification SEM observation of Pt-doped tin oxide, small crystallites at the surface of the spheres can be observed (Figure 5). Platinum doping might promote Ostwald ripening, a process where smaller nanoparticles dissolve and redeposit material onto larger particles (Sharma et al., 2019). This can lead to uneven surface roughness as nanoparticles of different sizes coalesce (Sharma et al., 2019).

**FIGURE 6:**
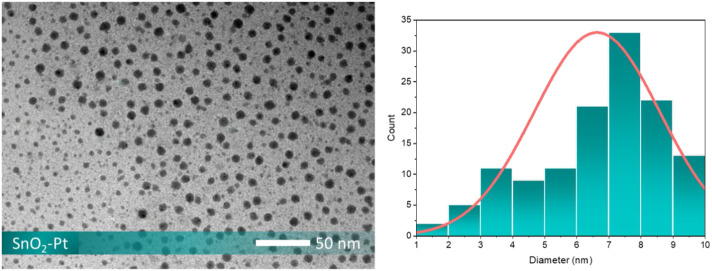
TEM image and diameter distribution of SnO_2_-Pt.

The effect of loading platinum onto the surface of the tin oxide it also confirmed by TEM investigation (Figure 6). Pt nanoparticles with an average size of 7∼8 nm are homogeneously dispersed onto the surface of SnO_2_ microspheres, and the spherical structure is made up of the aggregated SnO_2_ nanocrystals. In detail, both small and large spherical particles are displayed in the TEM image. The size variation in the lower-left of the image suggests imaging artifacts, sample thickness differences, or particle orientation. Our analysis of particle size distribution from 20 TEM images, evaluated using ImageJ software, showed kurtosis of 2.6, indicating a platykurtic distribution with a flatter shape and lighter tails that could explain the observed differences in particle sizes.

The specific surface area and pore-size distribution of the as-synthesized and doped SnO_2_ nanospheres are investigated by nitrogen adsorption–desorption isotherms. The pristine and loaded samples show the same adsorption behavior corresponding at type IV isotherm of the IUPAC classification (results not shown). In addition, the BET surface area values are very similar, respectively, 83.76 m^2^/g for pristine tin oxide and 84.01 m^2^/g for the platinum-loaded sample.

### Gas sensing

**FIGURE 7:**
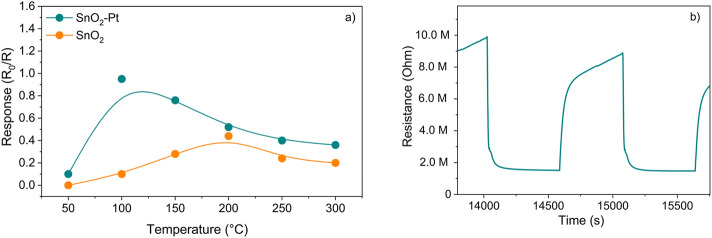
**(a)** SnO_2_ and SnO_2_-Pt sensor responses *versus* temperature at 0.5 mg/L of aqueous 2,3-butanedione solution; **(b)** typical transient response of SnO_2_-Pt sensor to 0.5 mg/L of aqueous 2,3-butanedione solution at 100°C.

The sensor working temperature was set at 100°C after considering the sensor response under a temperature screening in the range 50°C–300°C at 0.5 mg/L of 2,3-butanedione concentration (Figure 7a).

As a metal–organic semiconductor (MOS) with n-type behavior, tin oxide is responsible for the oxidation of the target gas over its surface, rich in oxygen species, which in turn causes the electrical resistance of the layer to decrease. The sensor demonstrates an n-type response, which signifies an electron-rich surface, and the sensor’s resistance R decreases upon exposure to the reducing analyte gas (such as 2,3-butanedione and/or ethanol) in comparison to the baseline sensor resistance R_0_ when subjected to the carrier flow. This indicates a response ratio denoted by R_0_/R. Conversely, in the oxidizing atmosphere, there is a noticeable increase in sensor resistance, resulting in R surpassing R_0_. The response is the opposite when an n-type sensor is exposed to reducing gases such as CO, CH_4_, H_2_S, NH_3_, and 2,3-butanedione (Sun et al., 2012), typically showing R < R_0_ resistance values. The doping of the oxide with 3% platinum maintained the n-type behavior but further increased its detection ability even at lower operating temperature with respect to a pure SnO_2_ sensor acting in similar experimental conditions (Gnisci et al., 2022).

As a general rule, the surface of SnO_2_ in air is coated with oxygen adsorbates that have a negative charge, and electrons are extracted from the conduction band of SnO_2_. This results in the formation of an electron depletion layer on the surface of the SnO_2_ grains, which are then fed back into the SnO_2_ bulk when the 2,3-butanedione molecules are oxidized by oxygen species on the surface as soon as the sensor meets with the analyte vapor and the resistance of the sensor decreases (Figure 7b).

**FIGURE 8:**
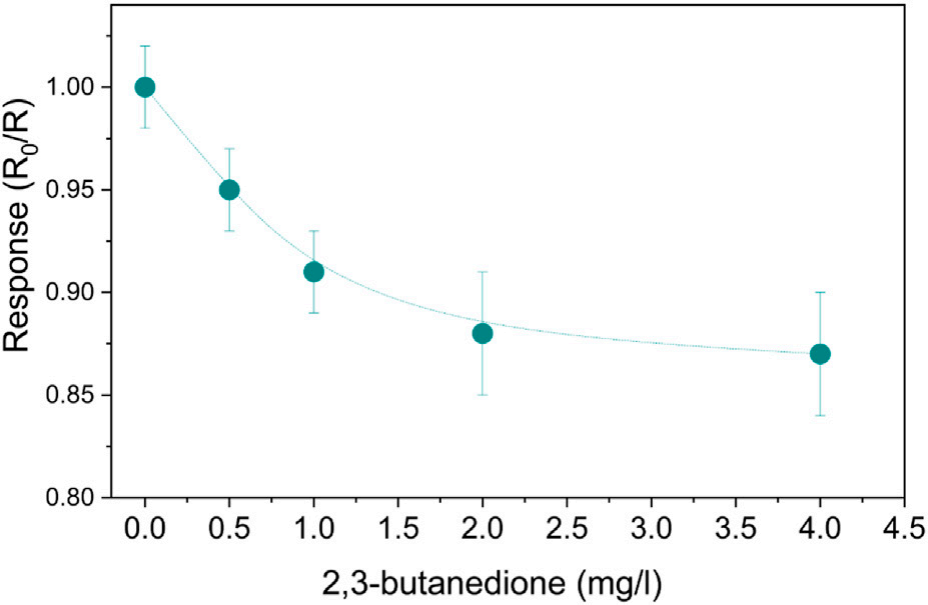
SnO_2_-Pt normalized responses to aqueous 2,3-butanedione solutions at 100°C.

An alcoholic fermentation ambient similar to human stomach conditions was reproduced. To investigate the SnO_2_-Pt sensor, the concentration of 2,3-butanedione was increased in aqueous solutions while sensing tests were carried out in a CO_2_ environment and the sensor regenerated in air conditions. The response curves for various concentrations of 2,3-butanedione, ranging from 0.5 to 4 mg/L, are presented in Figure 8. By increasing the concentration of 2,3-butanedione, it is possible to observe that the response of the sensor decreases, following an exponential trend. Despite the traditional n-type response, the sensing mechanism is basically due to the direct adsorption of 2,3-butanedione molecules, and generally VOCs, on the MOS surface (Abokifa et al., 2018; Bonaccorsi et al., 2022); here the oxidation is rapid thanks to the availability of oxygen sites but limited due to the absence of oxygen atmosphere that could further generate active sites. Therefore, the mere adsorption of unreacted analyte is responsible for the sensor resistance increment.

**FIGURE 9:**
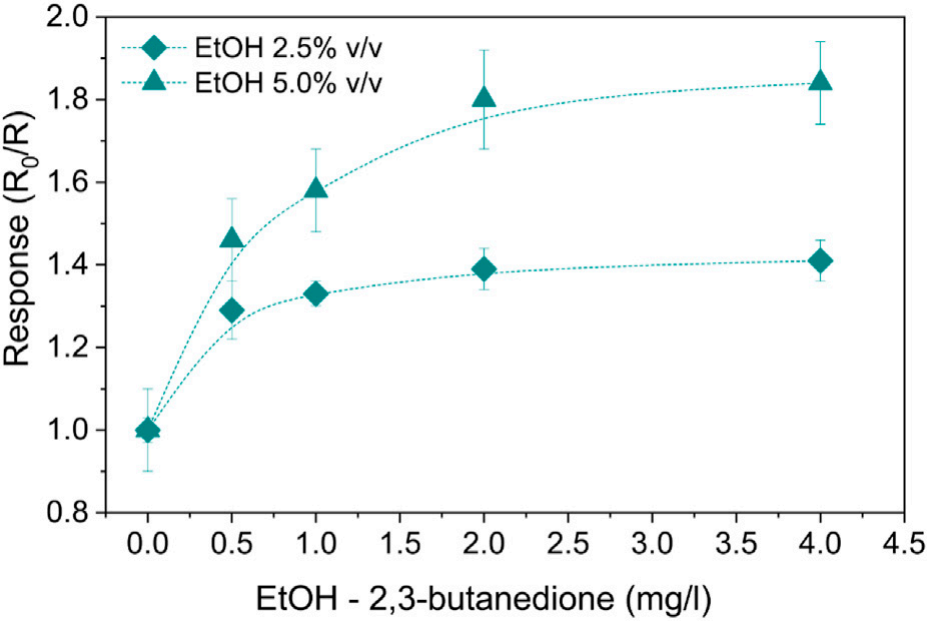
SnO_2_-Pt normalized responses to alcoholic 2,3-butanedione solutions with ethanol concentrations of 2.5% and 5.0% v/v at 100°C.

To consider the most interfering species produced in the human stomach, alcoholic 2,3-butanedione solutions obtained with the addition of two different ethanol concentrations—2.5% v/v and 5% v/v—were tested. A summary of the response values can be found in Figure 9. The sensing mechanism of 2,3-butanedione is similar, but the response is, in these cases, enhanced by the presence of ethanol, whose detection by SnO_2_ is well known in the literature due to the high sensitivity of this MOS toward ethanol (Maekawa et al., 1992). Indeed, the oxidation of ethanol in an anaerobic atmosphere has hydrogen as a by-product which, in turn, also participates in the oxidation process (Sobolev et al., 2012). The resulting electrons will return to the conduction band of the semiconductor, contributing to decreasing the resistance R. In particular, as a molecule of hydrogen is less awkward than 2,3-butanedione, its oxidation is not only limited to the surface oxygen species of the sensor but also involves in-depth structured oxygen.

Finally, when restored to the air environment, the sensor returns to its original state and can be used again.

Moreover, the two different ethanol concentrations similarly influenced the detection of 2,3-butanedione, resulting in respective responses proportional to each other (Figure 9).

Following exposure to the analyte gas, the sensor’s response/recovery time—a crucial parameter in sensing technology—delineates the duration needed for the sensor to reach 90% of its saturation limit and 10% of its original value within the carrier gas flow.

**FIGURE 10:**
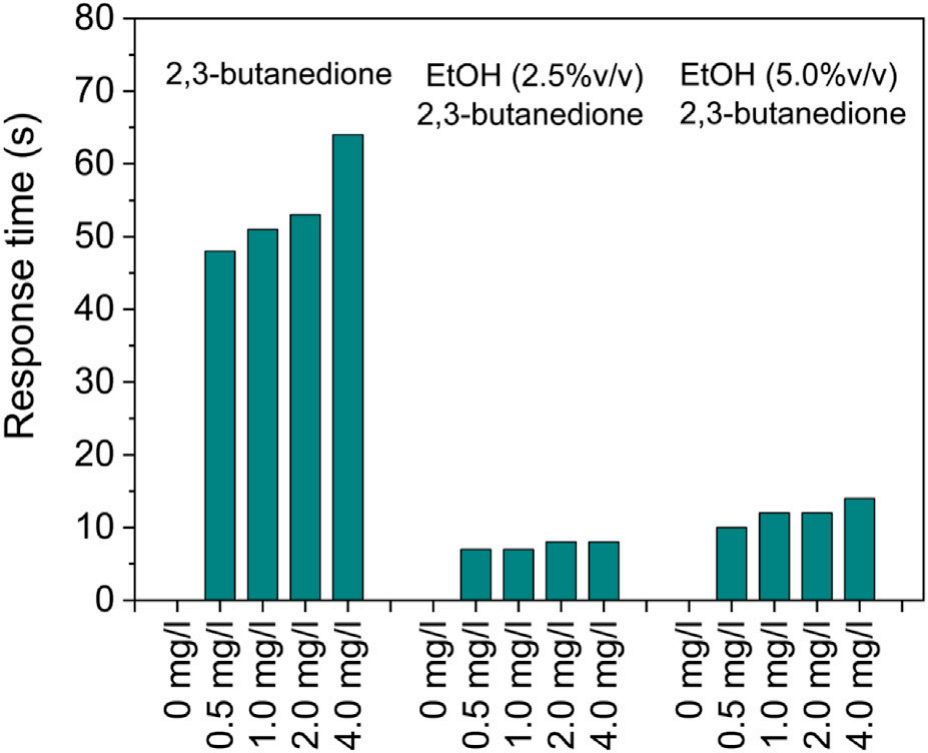
SnO_2_-Pt response times to pure and alcoholic 2,3-butanedione solutions at 100°C.

The sensor exhibited moderate transient responses (Figure 10), taking less than 60 s to attain 90% of the final signal in 2,3-butanedione solutions. Furthermore, responsiveness seems to be highly increased when alcoholic solutions are considered, resulting in less than 10 s. However, comparing the response to the recovery signals after removing the target gas, it is evident that the sensor exhibits very different behavior even if it is still capable of restoring the initial signal in a reasonably short period of time, despite being increased to 6 min.

## Conclusion

SnO_2_-Pt particles with spherical morphology were successfully synthesized via a simple hydrothermal route of SnO_2_ impregnation with a platinum source, followed by heat treatments.

For the first time, it has been demonstrated that surface-modifying SnO_2_ with Pt nanoparticles transforms this sensing material into a sensor with high sensitivity, fast response, and excellent selectivity to 2,3-butanedione.

An alcoholic fermentation environment similar to human stomach conditions was replicated to explore the SnO_2_-Pt sensor. Concentrations of 2,3-butanedione were elevated in aqueous solutions for sensing tests conducted in a CO_2_ environment, with sensor regeneration carried out in air conditions. By varying 2,3-butanedione concentrations (0.5–4 mg/L), it was observed that the sensor response diminishes with increased 2,3-butanedione concentration.

The sensor showed moderate transient responses, reaching 90% of the final signal in 2,3-butanedione solutions in under 60 s. Notably, responsiveness significantly improved in alcoholic solutions, achieving this level in less than 10 s. Although the sensor does recover the initial signal relatively quickly (within around 6 min), its behavior differs noticeably from the response phase after target gas removal. However, despite Pt nanoparticles enhancing the gas-sensing properties of SnO_2_ by improved catalytic activity, the increase of the surface area, and the facilitation of charge transfer, the high cost of platinum does present a significant economic barrier to sensor production. This is especially so for large-scale applications, which may hinder accessibility and widespread adoption in various industries. Additionally, it should be noted that the environmental impact of platinum extraction and processing raises sustainability concerns due to their substantial ecological footprints.

This research aimed to develop a sensing layer capable of detecting 2,3-butanedione, a compound known for its safety and potential health impacts. By synthesizing, characterizing, and testing platinum-decorated nanoparticles on a tin oxide semiconductor layer, the study has successfully laid the foundation for a simple, cost-effective, and rapid on-site detection layer for 2,3-butanedione. To advance the development and adoption of Pt-based sensors, further investigation should be addressed to overcome the aforementioned limitations and align with the increasing demand for sustainable technologies.

## Data Availability

The raw data supporting the conclusions of this article will be made available by the authors, without undue reservation.
